# Integrating polygenic risk scores in the prediction of gestational diabetes risk in China

**DOI:** 10.3389/fendo.2024.1391296

**Published:** 2024-08-06

**Authors:** Jiayi Cheng, Chan Meng, Junwei Li, Ziwen Kong, Aifen Zhou

**Affiliations:** ^1^ Department of Obstetrics, Wuhan Children’s Hospital (Wuhan Maternal and Child Health care Hospital), Tongji Medical College, Huazhong University of Science and Technology, Wuhan, China; ^2^ Institute of Maternal and Child Health, Wuhan Children’s Hospital (Wuhan Maternal and Child Health care Hospital), Tongji Medical College, Huazhong University of Science and Technology, Wuhan, China

**Keywords:** gestational diabetes mellitus, genome-wide association study, polygenic risk score, non-invasive prenatal testing, single nucleotide polymorphism

## Abstract

**Background:**

Polygenic risk scores (PRS) serve as valuable tools for connecting initial genetic discoveries with clinical applications in disease risk estimation. However, limited studies have explored the association between PRS and gestational diabetes mellitus (GDM), particularly in predicting GDM risk among Chinese populations.

**Aim:**

To evaluate the relationship between PRS and GDM and explore the predictive capability of PRS for GDM risk in a Chinese population.

**Methods:**

A prospective cohort study was conducted, which included 283 GDM and 2,258 non-GDM cases based on demographic information on pregnancies. GDM was diagnosed using the oral glucose tolerance test (OGTT) at 24–28 weeks. The strength of the association between PRS and GDM odds was assessed employing odds ratios (ORs) with 95% confidence intervals (CIs) derived from logistic regression. Receiver operating characteristic curves, net reclassification improvement (NRI), and integrated discrimination improvement (IDI) were employed to evaluate the improvement in prediction achieved by the new model.

**Results:**

Women who developed GDM exhibited significantly higher PRS compared to control individuals (OR = 2.01, 95% CI = 1.33–3.07). The PRS value remained positively associated with fasting plasma glucose (FPG), 1-hour post-glucose load (1-h OGTT), and 2-hour post-glucose load (2-h OGTT) (all *p* < 0.05). The incorporation of PRS led to a statistically significant improvement in the area under the curve (0.71, 95% CI: 0.66–0.75, *p* = 0.024) and improved discrimination and classification (IDI: 0.007, 95% CI: 0.003–0.012, *p* < 0.001; NRI: 0.258, 95% CI: 0.135–0.382, *p* < 0.001).

**Conclusions:**

This study highlights the increased odds of GDM associated with higher PRS values and modest improvements in predictive capability for GDM.

## Introduction

Gestational diabetes mellitus (GDM) is characterized by glucose intolerance that arises during pregnancy and typically resolves after delivery (1). In China, the incidence of GDM is alarmingly high, estimated at 14.8% (2), and this figure continues to rise annually. Studies have indicated that abnormal increases in blood glucose levels are strongly associated with an increased risk of adverse health outcomes for both mothers (3–6) and their offspring (7) during pregnancy and later in life (8–10). Therefore, identifying risk factors associated with GDM before or early in pregnancy is crucial for improved monitoring, the development of safe and timely interventions, and treatments.

GDM is a complex disorder influenced by a combination of demographic, genetic, and environmental factors, resulting in ethnic differences in its occurrence. The genetic variants for disease characteristics have identified rapidly in the past decade. Genome-wide association studies (GWAS) have provided summary statistics that describe the effect size and statistical significance of the association between specific alleles and disease outcomes (11). Some studies have elucidated genetic variants associated with type 2 diabetes in both Caucasians and Asians, revealing some similarities in the genes associated with the disease (12, 13). For example, certain genes implicated in type 2 diabetes, such as *CDKAL1*, *IGF2BP2*, *TCF7L2*, *KCNQ1*, and *MTNR1B*, have been associated with the risk of GDM (14). Among Korean populations, *MTNR1B* and *CDKAL1* have been shown to exhibit positive associations with GDM risk (15, 16).

However, recognizing that assessing the risk of a complex disease such as GDM cannot solely rely on a single genetic variant is essential. In recent years, PRSs have been developed for a range of health traits and conditions. These scores, which are based on summary statistics from GWAS, have been developed as an innovative approach to evaluating the genetic risk of a disease by considering the cumulative effects of multiple genetic loci. This approach can be used to stratify individuals based on their genetic risk of acquiring various diseases, improving screening, preventative interventions, and patient care (17). Some research has shown the promise of PRSs in identifying genetic risk for various diseases, including Alzheimer’s disease and cardiovascular diseases (18–20). Ho et al. found that the predictability of PRSs varies across different diseases (21). While the degree of predictability for individual disease risk using PRSs remains different, emerging data supports the utilization of PRSs for population-based GDM. Therefore, in this study, we implemented this novel approach to assess the genetic risk of GDM.

GDM is typically diagnosed based on the results of an oral glucose tolerance test (OGTT) performed between 24 and 28 weeks of pregnancy. However, the damage induced by abnormal blood glucose levels in early pregnancy cannot be reversed. Therefore, herein, we conducted a prospective cohort study on a Chinese population to investigate the predictive validity of the PRS for GDM among pregnant women and developed a prediction model that incorporates both traditional and genetic factors associated with GDM. By screening for these factors, we aimed to enhance the identification of women at risk of developing GDM, enabling early intervention and the implementation of management strategies.

## Methods

### Study population

**Figure 1:**
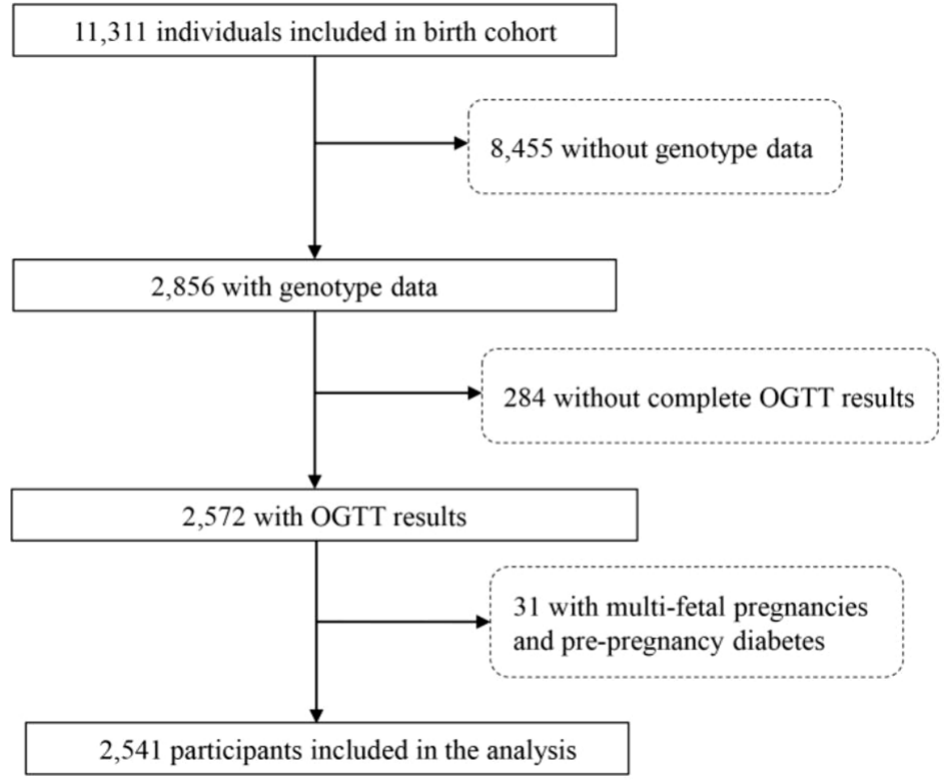
The flow chart of population inclusion criteria.

The present study was conducted between October 2012 and September 2019 at Wuhan Women and Children’s Medical Care Center. A total of 11,311 pregnant women who met the following criteria were recruited: a) < 16 wk of pregnancy with a singleton gestation at the time of enrollment; b) resident of Wuhan City; c) willing to have prenatal care and give birth at the study hospital. (**Figure 1**). The study protocol was approved by the ethics committee of Tongji Medical College, Huazhong University of Science and Technology (number (2012)14) and the Wuhan Women and Children Medical and Healthcare Center (number 2010009). All participants in this study provided written informed consent prior to participation.

### Definition of GDM

GDM was defined according to the diagnostic criteria of the International Association of Diabetes and Pregnancy Study Group (IADPSG) and local policy. Universal testing for GDM was conducted between the 24^th^ and 28^th^ weeks of gestation, employing the 75 g 2-h OGTT. GDM was diagnosed if fasting glucose was ≥5.5 mmol/L, 1-h glucose was ≥10.0 mmol/L, and/or 2-h glucose was ≥8.0 mmol/L. Based on these criteria, a total of 265 participants in the cohort were initially diagnosed with GDM during pregnancy.

### Covariates

Demographic information of the participants was collected using questionnaires. The collected data encompassed maternal education level, economic status, smoking status, pre-pregnancy body mass index (PBMI), age, alcohol consumption during pregnancy, house decoration, employment status, physical activity, gravidity, and parity. Maternal educational level (≤9/9–12/≥12 schooling years), economic status (very good/good/normal/poor/very poor), physical activity (never or rarely, 1–2 days/week, 3–4 days/week, 5–6 days/week, daily), smoking status (yes/no), alcohol consumption (yes/no), employment status (yes/no), multiparity (yes/no), gravidity (1/2/≥3), and decorating status (yes/no) were among the variables considered. PBMI was calculated by dividing pre-pregnancy weight (in kg) by height squared (in m^2^).

### Genotype data and calculation of PRS

Genotype data were obtained using peripheral venous blood samples collected during non-invasive prenatal testing (NIPT) screening (22). Next-generation high-throughput sequencing was performed at BGI-Wuhan, yielding ultra-low sequencing depth ranging from 0.06× to 0.1× per individual. The BaseVar algorithm was used to call single nucleotide polymorphisms (SNPs), and genotype imputation was performed using STITCH. On the basis of this theory, we excluded samples with sequencing depth <0.05× or mapping rates <90% from the analysis. The imputation accuracy was assessed by randomly selecting 30 Han Chinese individuals from the 1,000 Genomes Project with a sequencing coverage of 30×. Their data were down-sampled to a sequencing depth of 0.1× and imputed using the true NIPT genotype data through STITCH. The imputation accuracy was evaluated by calculating Pearson’s correlation coefficient between the true genotype set (at 30× coverage) and the imputed genotype. The imputed genotypes were represented as dosages ranging from 0 to 2. For further analysis, biallelic variants with a minor allele frequency (MAF) >0.05, a Hardy–Weinberg equilibrium (HWE) *p*-value >1e-6, and a genotype missing rate <0.1 were included. Genotype dosages were used to compute the PRS for GDM.

The calculation of PRS was based on multi-ancestry GWAS summary statistics (23). Our study utilized a multi-ancestry meta-analysis with an effective sample size comprising 72.2% European, 13.4% East Asian, 9.9% South Asian, 2.8% Hispanic/Latino, and 1.7% African participants. This analysis identified five SNPs strongly associated with GDM, including the previously reported GWAS for *MTNR1B* (rs10830963, *p* = 4.3 × 10^−54^) and *CDKAL1* (rs9348441, *p* = 1.6 × 10^−14^). The remaining three loci for GDM mapped to/near *TCF7L2* (rs7903146, *p* = 4.0 × 10^−16^), *CDKN2A-CDKN2B* (rs10811662, *p* = 4.1 × 10^−9^), and *HKDC1* (rs9663238, *p* = 2.9 × 10^−8^). To calculate the PRS for each individual in this study, we identified the risk alleles they carried and used the effect size of each risk allele to weight the calculation. To ensure comparability across individuals, we standardized all PRS to have a mean of zero and a unit variance.

### Statistical analyses

Statistical analysis was performed using SPSS version 19.0. Continuous variables were expressed as the mean ± standard deviation (SD) and analyzed using the independent sample Student’s t-test. Categorical variables were described as frequencies and percentages, with their analysis employing the chi-square test or Fisher’s exact test, as appropriate. Variables found to be associated with GDM in univariate analysis (*p* < 0.05) and those previously documented in the literature were incorporated into the analysis. A correlation analysis was conducted to examine the relationship between the values of oral glucose tolerance and the PRS. The PRS was treated as a categorical variable in the models, with each quintile of the PRS evaluated for its association with GDM. A multivariate logistic regression model was fitted to estimate odds ratios (ORs), with associated 95% confidence intervals (CIs) to predict GDM. GDM status served as the outcome variable, with maternal traditional risk factors and PRS employed as predictors. Due to the final dataset comprising 283 cases (11.1%) and 2,258 cases (88.9%), which is extremely imbalanced, we calculated the average of the model evaluation metrics through five-fold cross-validation and the incorporation of under-sampling. Receiver operating characteristic (ROC) curves were used to estimate the area under the curve (AUC), net reclassification improvement (NRI), and integrated discrimination improvement (IDI) to compare two prediction models: the traditional risk factors model and the traditional risk factors model with PRS.

## Results

### Characteristics of the study population

**Table 1** presents the characteristics of the 2,541 participants included in the study, with 283 participants (11.1%) diagnosed with GDM. Significant differences in maternal age and PBMI were observed between the GDM and non-GDM groups. In the GDM group, the average age was 30 ± 4 years, and the mean PBMI was 22.8 ± 3.5 kg/m^2^. Most neonates were born to primiparous mothers (n = 217, 76.7%). The prevalence of smoking and alcohol consumption during pregnancy was low, with most mothers reporting non-smoking (n = 172, 60.8%) and abstinence from alcohol (n = 278, 98.1%). Additionally, approximately half of the women were employed during pregnancy (n = 161, 56.9%), and most mothers in the GDM group received a general income (n = 195, 68.9%).

**Table 1:** Demographic characteristics of participants in the GDM group and the non-GDM group.

		Non GDM (n=2258)	GDM (n=283)	*p* value
Pre-pregnancy BMI (kg/m2)		21.00±2.82	22.80±3.59	<0.01^a^
Age (year)		28.99±3.54	30.46±3.94	<0.01^a^
Gravidity (%)				
	1	1402 (62.1%)	140 (49.5%)	<0.01^b^
	2	477 (21.1%)	81 (28.6%)	
	≥3	379 (16.8%)	62 (21.9%)	
Parity (%)				
	1	1852 (82.0%)	217 (76.7%)	0.026^b^
	≥2	406 (18.0%)	66 (23.3%)	
House decoration (%)				
	No	1400 (62.0%)	195 (68.9%)	0.028^b^
	Yes	858 (38.0%)	88 (31.1%)	
Smoking status (%)				
	No	1452 (64.3%)	172 (60.8%)	0.272^b^
	Yes	806 (35.7%)	111 (39.2%)	
Physical activity (a week,%)				
	0	252 (11.2%)	29 (10.2%)	0.091^b^
	1-2	216 (9.6%)	30 (10.6%)	
	3-4	124 (5.5%)	11 (3.9%)	
	5-6	62 (2.7%)	1 (0.4%)	
	7	1604 (71%)	212 (74.9%)	
Alcohol consumption (%)				
	No	2192 (97.1%)	278 (98.1%)	0.346^b^
	Yes	66 (2.9%)	5 (1.9%)	
Employment status (%)				
	No	849 (37.6%)	122 (43.1%)	0.043^b^
	Yes	1409 (62.4%)	161 (56.9%)	
Maternal education level (years of schooling,%)				
	≤9	122 (5.4%)	17 (6%)	0.817^b^
	9-12	275 (12.2%)	36 (12.8%)	
	≥12	1861 (82.4%)	230 (81.1%)	
Economic status (%)				
	Very good	56 (2.5%)	13 (4.6%)	0.072^b^
	good	736 (32.6%)	73 (25.8%)	
	normal	1454 (64.4%)	195 (68.9%)	
	poor	8 (0.4%)	1 (0.4%)	
	very poor	4 (0.2%)	1 (0.4%)	
Pre-eclampsia (%)				
	No	2248 (99.6%)	279 (98.6%)	0.098
	Yes	10 (0.4%)	4 (1.4%)	
Cholestasis (%)				
	No	2254 (99.8%)	281 (99.3%)	0.279
	Yes	4 (0.2%)	2 (0.7%)	
Other disease (%)				
	No	2251 (99.7%)	280 (98.9%)	0.163
	Yes	7 (0.3%)	3 (1.1%)	

Decorate: decorate the house in recent 3 years.

Values are presented as numbers (percentages) or means ± SD, as appropriate. ^a^t-test. ^b^chi-square test or Fisher's exact test.

### Association between PRS and GDM

**Figure 2:**
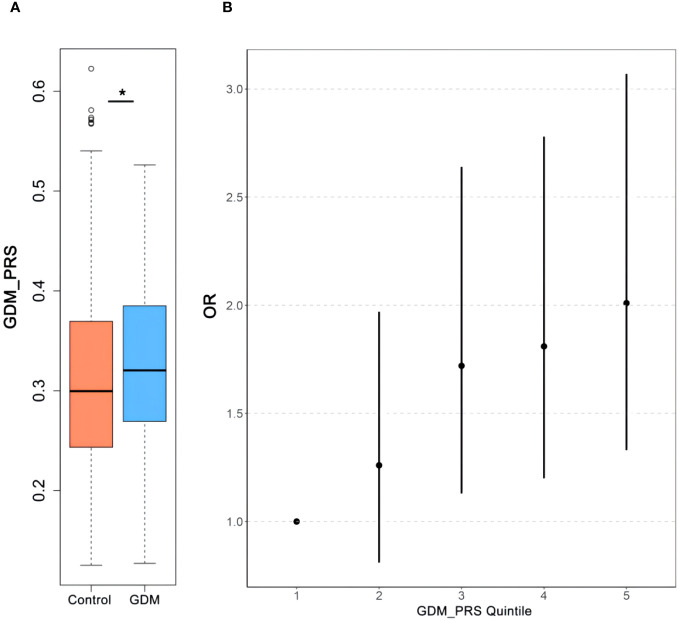
The association between PRS to GDM. Comparisons of the GDM-PRS **(A)** of the subjects with GDM and Control. Relationship of GDM-PRS quintile **(B)** with GDM. *p<0.05. PRS, polygenic risk score; GDM, gestational diabetes mellitus.

**Figure 3:**
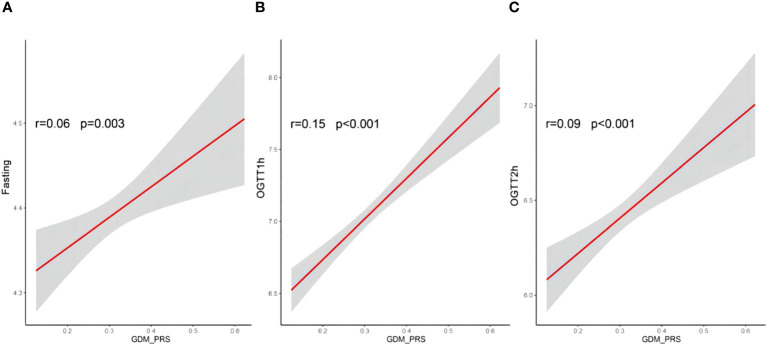
Correlations between PRS of GDM and fasting **(A)**, OGTT1h **(B)** and OGTT2h **(C)** glucose. Pearson correlation coefficients (r) were used to assess the correlatios; PRS, polegenic risk score; OGTT, oral glucose tolerance test results; GDM, gestational diabetes mellitus.

Logistic regression analyses were conducted to assess the relationship between PRS and GDM. The ORs with their corresponding 95% CIs for GDM across increasing quintiles of PRS levels were as follows: 1.00 (reference), 1.26 (0.81–1.97), 1.72 (1.13–2.64), 1.81 (1.20–2.78), and 2.01 (1.33–3.07) (**Figure 2**). Women who developed GDM exhibited significantly higher PRS than the control individuals (mean ± SD: 0.33 ± 0.08 in the GDM group, 0.31 ± 0.08 in the non-GDM group, *p* < 0.05). Significant positive correlations were observed between PRS and fasting plasma glucose(FPG), 1-h OGTT, and 2-h OGTT (**Figure 3**). The distribution of traditional risk factors based on the quintile classification of the PRS is shown in **Supplementary Table S1**.

### Correlations between traditional risk factors and GDM

Significant differences were observed between gravidity, parity, employment during pregnancy, and house decoration in the past 3 months, indicating their association with GDM (*p* < 0.05). However, educational level, smoking, alcohol consumption, income, and physical activity did not show statistically significant differences (*p* > 0.05). Variables exhibiting statistical significance were included in the multivariate logistic regression analysis.

### Multivariate logistic regression analysis

As shown in **Table 2**, the multivariate logistic regression analysis indicated that PBMI (OR = 1.14, *p* < 0.001), maternal age (OR = 1.09, *p* < 0.001), employment during pregnancy (OR = 0.75, *p* = 0.039), gravidity (OR = 1.43, *p* = 0.042), and PRS (OR = 19.68, *p* < 0.001) were independent risk factors influencing the occurrence of GDM. Lower PBMI and younger age were identified as significant protective factors against GDM. These results suggest that PRS, along with traditional risk factors such as PBMI, maternal age, employment during pregnancy, and gravidity, play an important role in the development of GDM.

**Table 2:** Multivariate regression analysis for the PRS and traditional risk factors.

	Odd ratio(95% CI)	*p* value
Pre-pregnancy BMI	1.14 (1.10-1.20)	<0.001
Age	1.09 (1.05-1.13)	<0.001
Employment status	0.75 (0.57-0.99)	0.039
Gravidity		0.115
2	1.43 (1.01-2.03)	0.042
≥3	1.36 (0.87-2.11)	0.174
Parity	0.66 (0.43-1.01)	0.055
GDM-PRS	19.68 (4.07-95.08)	<0.001
Constant		<0.001

SE, standard error; 95% CI, 95% confidence interval. Odds ratios indicate the relational size of effect arising from the specific variable class listed (for categorical variables) or one-unit change (for continuous variables).

### Predictive performance of the model

**Figure 4:**
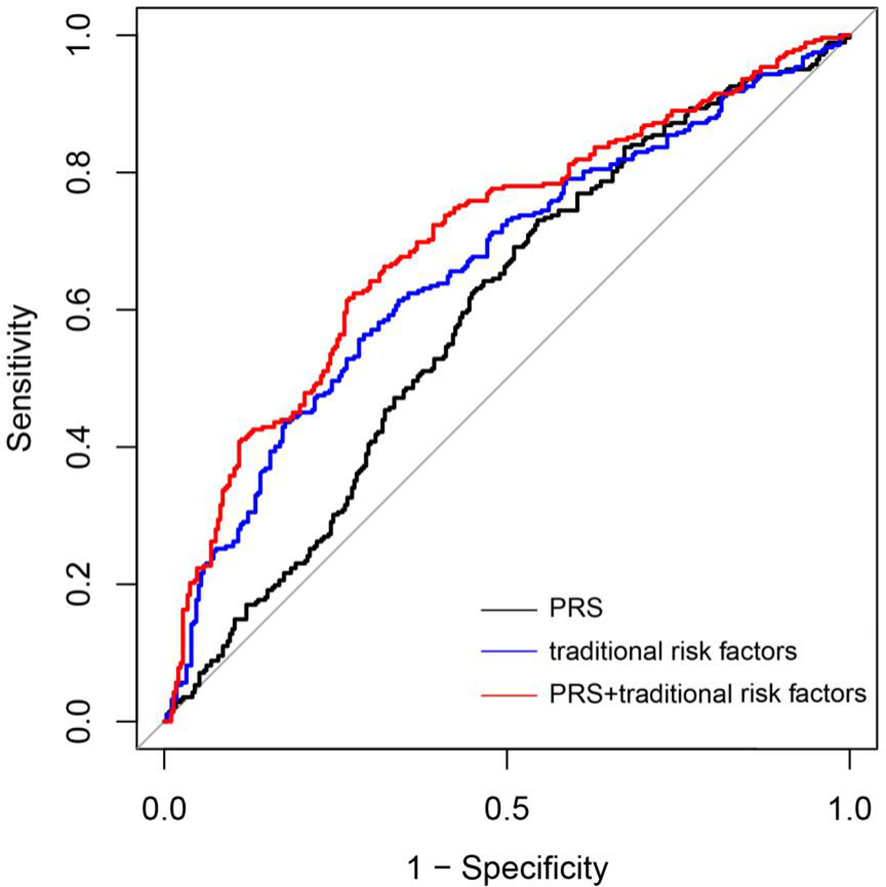
Accuracy of predicting GDM using a PRS and tradional risk factors. ROC curves of the novel prediction model in comparison to a PRS model and Traditional risk factors model. PRS mode 1 (black line); Traditional risk factors model: Pre-pregnancy BMO, Age, Working condition and Gravidity (blue line); Combined traditional risk factors and PRS model: Pre-pregnancy BMI, Age, Working condition and Gravidity and PRS (red line).

The model’s predictive performance was assessed employing the ROC curve (**Figure 4**). The ROC curve illustrates the discriminative abilities of different models. The basic model, which included only PRS (Model 1), yielded an AUC of 0.59 (95% CI: 0.54–0.64). When traditional risk factors, such as maternal age, parity, gravidity, PBMI, and employment status, were added to the model (Model 2), the AUC improved to 0.66 (95% CI: 0.62–0.71). Furthermore, the incorporation of PRS into the model with traditional risk factors (Model 3) further increased the AUC to 0.71 (95% CI: 0.66–0.75, *p* = 0.024). However, AUC as an evaluation metric may not be sensitive enough in certain scenarios, failing to adequately capture subtle changes or differences in model performance. To substantiate our claims more robustly, we incorporated the NRI and IDI indices into our analysis. The NRI and the IDI of the combined traditional risk factor model and traditional risk factors with PRS were calculated using the R language. The incorporation of PRS into the traditional risk factor model improved the discrimination and classification (IDI: 0.007, 95% CI: 0.003–0.012; NRI: 0.258, 95% CI: 0.135–0.382) of GDM. These results indicate that the improvement in the NRI for the GDM group is meaningful (0.2043, 95% CI: 0.0812–0.3274), while the improvement in the NRI for the non-GDM group did not reach statistical significance (0.0457, 95% CI: -0.0774–0.1688). These results support our conclusion that PRS can effectively augment the predictive accuracy of GDM risk models.

## Discussion

In this prospective cohort study, a notable and positive association was identified between PRS and GDM. PRS also exhibited positive correlations with FPG, 1-h OGTT, and 2-h OGTT. Although the correlation coefficient was small but significant, it potentially indicates a real but weak association between PRS and GDM. The magnitude and significance of the correlation coefficient can be influenced by multiple factors, including sample size, data distribution, and measurement errors. Consequently, further large-sample studies are required to validate its reliability and generality. Furthermore, we observed that incorporating PRS into the prediction model mildly improved the prediction of GDM.

Although individual SNPs are not sufficient to accurately predict disease risk, PRS allows for an assessment of overall genetic risk. PRS quantifies genetic factors by incorporating diverse low-penetrance variants identified through GWAS. By combining these associations, PRS provides a measure of genetic predisposition to a heritable trait, which can be used for disease risk stratification, prognostic predictions, and personalized prevention strategies (17, 24). Previous studies have demonstrated the utility of PRS in predicting disease risk and guiding preventive measures (25). These scores have already been demonstrated to predict breast cancer, lung cancer, prostate cancer, and post-transplant diabetes mellitus risk in patients of European descent more accurately compared to current clinical models (21, 26, 27). Our study indicates that individuals in the fifth quintile exhibit a 2.01-fold higher risk of developing diabetes compared with those in the first quintile. These findings are consistent with those of a previous GWAS on GDM. Our study validated the associations between PRS and GDM risk, indicating that women with a higher PRS may exhibit high fasting glucose concentrations or abnormal expression of glucose metabolism-related genes, leading to insulin resistance and an increased risk of GDM. Another study supports our findings, demonstrating a strong association between the PRS and GDM risk (16). The PRS derived from their optimal model indicated a significantly higher risk of GDM (28). Collectively, these findings underscore the importance of incorporating genetic risk factors, as captured by the PRS, in predicting GDM.

In this study, we conducted a comparative analysis of general characteristics and anthropometric measurements between pregnant women in the GDM and non-GDM groups. Several risk factors for GDM were identified, including PBMI, age, gravidity, and employment status. These findings are consistent with those of previous research that has also identified older age, higher BMI, and multigravidity as risk factors for GDM (29, 30). However, we did not observe significant associations with smoking, alcohol consumption, house decoration, income, and physical activity in the multivariate analysis. These results differ from those of a previous study conducted on a different population (31). These discrepancies may be attributed to differences in the study population or other confounding factors.

Previous predictive models have explored the potential of combining biomarkers to reduce the prevalence of maternal disease and adverse pregnancy outcomes (29, 32). One notable strength of our model is the incorporation of genetic factors, in contrast to other published multivariate risk prediction models (33, 34). Ideally, the selected genetic variants that constitute PRS should be relevant to the population being screened. While the PRS demonstrated an association with increased GDM risk in this study, its utility in predicting GDM cases was limited. This is consistent with recent studies indicating that the predictive performance of polygenic scores for complex traits, including GDM, has been shown to be modest in clinical settings (35). To date, all studies employing robust GWAS to assess the predictive value of PRS across a range of traits and populations have consistently reported the same observation: PRS predicts individual risk far more accurately in Europeans than in non-Europeans (36–38). Most GWAS have primarily focused on European populations, resulting in a bias towards this specific group in multi-ancestry GWAS meta-analyses (39). Additionally, differences in allele frequencies and linkage disequilibrium patterns between populations can render accurately identifying the causal variant challenging, thereby limiting the predictability of multi-ancestry meta-analyses in continental Chinese populations (39, 40). Furthermore, factors such as diverse environmental exposures, gene–gene interactions, gene–environment interactions, historical population dynamics, statistical noise, and potential causal effect differences further restrict the generalizability of genetic risk scores (41). The development of a PRS model based on SNPs identified within the Chinese population would likely provide a more accurate prediction of the genetic risk of GDM in Chinese individuals. However, it should be noted that the reported AUCs may vary owing to differences in the prevalence of GDM and testing criteria across studies (42). Although AUC is used to evaluate the model’s ability to distinguish between final events, it exhibits a certain value; however, it is not sensitive to changes in absolute risk estimation and lacks specific clinical significance (43). Whether the use of AUC is a sensitive enough metric to assess the clinical utility of polygenic prediction has remained a subject a debate (44, 45). Therefore, we further employed NRI and IDI (43) to assess the improvement in risk prediction accuracy achieved by the new model compared to traditional risk factor models. The results showed that the prediction model with PRS improved the proportion of correctly reclassified individuals and overall discrimination ability compared to the traditional risk factor models, with statistical significance.

GDM is influenced by a combination of genetic and environmental factors, each of which has the potential to contribute to its onset (23). Our study has several strengths, including being the first to systematically investigate the association between PRS and GDM with a relatively large sample size. We optimized PRS for our specific population and used objective OGTT measures to determine GDM status. The genetic approach to predicting GDM holds the advantage of being applicable early in pregnancy, which will allow for intervention before any adverse outcomes associated with hyperglycemia occur.

Nonetheless, our study has certain limitations that warrant acknowledgment. Firstly, our current predictive model was derived from a cohort study and included several known risk factors for GDM, such as age, PBMI, and gravidity. Besides demographic factors, future studies may incorporate other risk factors, such as clinical characteristics (32) and family history of the disease (46, 47), to further enhance the efficacy of the predictive model. Secondly, in this study, PRS were based on SNPs identified in other ethnicities. Given the potential variations in the risk loci of GDM among ethnic groups, currently few loci have been found to be associated with the incidence of GDM. A PRS model based on SNPs identified in the Chinese population would offer a more accurate prediction of the genetic risk of GDM in Chinese individuals. The exploration of genetic variants specific to the Chinese population would enhance the precision of GDM risk prediction in this demographic. Thirdly, external validation of our novel prediction model is required to assess its generalizability and performance in different populations in China. The interaction between traditional risk factors and genetic factors should also be further explored and validated. Lastly, this study lacked information on lifestyle factors, such as food intake during pregnancy (48). These factors can interact with the PRS for GDM risk and may influence the overall risk. Future studies should consider incorporating comprehensive lifestyle data to better understand the effects of these factors on GDM risk.

## Conclusion

In the present study, the PRS had significant correlations with GDM in our cohort, the use of a PRS has shown promise in predicting the risk of GDM and has the potential to be corporated into personalized. As genetic risk profiles vary among populations, large-scale genome-wide sequencing studies are urgently needed to identify the genetic risk loci of GDM in Chinese populations to build accurate PRS models for clinical practice.

## Data availability statement

The raw data supporting the conclusions of this article will be made available by the authors, without undue reservation.

## Ethics statement

The studies involving humans were approved by the Wuhan Children’s Hospital (Wuhan Maternal and Child Health care Hospital), Tongji Medical College, Huazhong University of Science and Technology, Wuhan, China.. The studies were conducted in accordance with the local legislation and institutional requirements. Written informed consent for participation was not required from the participants or the participants' legal guardians/next of kin in accordance with the national legislation and institutional requirements. Written informed consent was obtained from the individual(s) for the publication of any potentially identifiable images or data included in this article.

## Author contributions

JC: Conceptualization, Data curation, Formal analysis, Investigation, Methodology, Project administration, Software, Writing – original draft, Writing – review & editing. CM: Resources, Software, Supervision, Visualization, Writing – review & editing. JL: Data curation, Formal analysis, Project administration, Software, Writing – original draft. ZK: Data curation, Software, Writing – original draft. AZ: Conceptualization, Formal analysis, Investigation, Methodology, Supervision, Writing – review & editing.
