# Supplementary material for: Integrating polygenic risk scores in the prediction of gestational diabetes risk in China
Source: Front Endocrinol (Lausanne). 2024 Aug 6;15:1391296. doi: 10.3389/fendo.2024.1391296 (PMC11333217; doi:10.3389/fendo.2024.1391296)
Supplement: Supplementary file 1 [file Table_1.docx]

**Table S1.** GDM-specific Polygenic Risk Score (GDM-PRS) quintiles for Patients’demographics characteristics.

| Characteristics |  | PRS of GDM | | | | |  |
| --- | --- | --- | --- | --- | --- | --- | --- |
|  |  | Q1(N=509) | Q2(N=508) | Q3(N=508) | Q4(N=508) | Q5(N=508) | *p* value |
| Pre-pregnancy BMI (kg/m2) |  | 21.15±2.91 | 21.25±2.93 | 21.35±3.01 | 21.24±3..03 | 21.010±2.96 | 0.432 |
| Age（year) |  | 29.22±3.61 | 29.44±3.66 | 29.22±3.70 | 29.03±3.50 | 28.85±3.59 | 0.098 |
| Gravidity(%) |  |  |  |  |  |  | 0.763 |
|  | 1 | 309 (60.7) | 309 (60.8) | 310 (61.0) | 298 (58.7) | 317 (62.4) |  |
|  | 2 | 105 (20.6) | 114 (22.4) | 116 (22.8) | 110 (21.7) | 112 (22.0) |  |
|  | ≥3 | 95 (18.7) | 85 (16.7) | 82 (16.1) | 100 (19.7) | 79 (15.6) |  |
| Parity(%) |  |  |  |  |  |  | 0.487 |
|  | 1 | 417 (81.9) | 405 (79.7) | 420 (82.7) | 406 (79.9) | 422 (83.1) |  |
|  | ≥2 | 92 (18.1) | 103 (20.3) | 88 (17.3) | 102 (20.1) | 86 (16.9) |  |
| Decorate(%) |  |  |  |  |  |  | 0.206 |
|  | No | 321 (63.2) | 325 (64.1) | 316 (62.3) | 298 (58.7) | 332 (65.6) |  |
|  | Yes | 187 (36.8) | 182 (35.9) | 191 (37.7) | 210 (41.3) | 174 (34.4) |  |
| Smoking status(%) |  |  |  |  |  |  | 0.319 |
|  | No | 322 (63.3) | 334 (65.7) | 307 (60.4) | 337 (66.3) | 326 (64.2) |  |
|  | Yes | 187 (36.7) | 174 (34.3) | 201 (39.6) | 171 (33.7) | 182 (35.8) |  |
| Physical activity(a week,%) |  |  |  |  |  |  | 0.936 |
|  | 0 | 56 (11.0) | 62 (12.2) | 53 (10.4) | 54 (10.6) | 54 (10.6) |  |
|  | 1-2 | 41 ( 8.1) | 52 (10.2) | 54 (10.6) | 47 ( 9.3) | 52 (10.2) |  |
|  | 3-4 | 32 ( 6.3) | 24 ( 4.7) | 25 ( 4.9) | 34 ( 6.7) | 19 ( 3.7) |  |
|  | 5-6 | 11 ( 2.2) | 12 ( 2.4) | 14 ( 2.8) | 14 ( 2.8) | 14 ( 2.8) |  |
|  | 7 | 369 (72.5) | 358 (70.5) | 362 (71.3) | 359 (70.7) | 369 (72.6) |  |
| Alcohol consumption(%) |  |  |  |  |  |  | 0.163 |
|  | No | 494 (97.1) | 500 (98.4) | 496 (97.6) | 487 (95.9) | 493 (97.0) |  |
|  | Yes | 15 ( 2.9) | 8 ( 1.6) | 12 ( 2.4) | 21 ( 4.1) | 15 ( 3.0) |  |
| Working condition(%) |  |  |  |  |  |  | 0.459 |
|  | No | 183 (36.0) | 184 (36.2) | 199 (39.2) | 200 (39.4) | 204 (40.2) |  |
|  | Yes | 326 (64.0) | 324 (63.8) | 309 (60.8) | 308 (60.6) | 304 (59.8) |  |
| Maternal education level(years of schooling,%) |  |  |  |  |  |  | 0.100 |
|  | ≤9 | 131 (25.7) | 122 (24.0) | 123 (24.2) | 106 (20.9) | 114 (22.4) |  |
|  | 9-12 | 259 (50.9) | 270 (53.1) | 256 (50.4) | 271 (53.3) | 259 (51.0) |  |
|  | ≥12 | 119 (23.4) | 116 (22.8) | 129 (25.4) | 131 (25.8) | 135 (26.6) |  |
| Economic status(%) |  |  |  |  |  |  | 0.931 |
|  | Very good | 15 ( 2.9) | 14 ( 2.8) | 9 ( 1.8) | 12 ( 2.4) | 16 ( 3.1) |  |
|  | good | 155 (30.5) | 161 (31.7) | 170 (33.5) | 168 (33.1) | 158 (31.1) |  |
|  | normal | 336 (66.0) | 331 (65.2) | 329 (64.8) | 324 (63.8) | 330 (65.0) |  |
|  | poor | 2 ( 0.4) | 1 ( 0.2) | 0 ( 0.0) | 3 ( 0.6) | 3 ( 0.6) |  |
|  | very poor | 1 ( 0.2) | 1 ( 0.2) | 0 ( 0.0) | 1 ( 0.2) | 1 ( 0.2) |  |
